# Real-Time Detection System for Road Roughness Based on Ultrasonic Technology

**DOI:** 10.3390/s26134324

**Published:** 2026-07-07

**Authors:** Hongjia Zhao, Libo Wang, Yimin Zhao, Xiaodong Sun

**Affiliations:** College of Communication Engineering, Jilin University, Changchun 130022, China; zhaohj2023@mails.jlu.edu.cn (H.Z.); wanglb9922@mails.jlu.edu.cn (L.W.); zhaoym2023@mails.jlu.edu.cn (Y.Z.)

**Keywords:** ultrasonic sensor, pavement friction coefficient, signal conditioning, fuzzy logic, real-time detection, intelligent transportation system

## Abstract

**Highlights:**

**What are the main findings?**

- An ultrasonic-based real-time detection method for road roughness is proposed, establishing the quantitative correlation between echo amplitude, road roughness and friction coefficient via fuzzy logic;
- The system achieves effective distance attenuation compensation, with a detection relative error of less than 10% compared with the standard pendulum tester on dry asphalt pavements.

**What are the implications of the main findings?**

- Road surface roughness is strongly correlated with the friction coefficient; measuring road surface roughness provides the foundation for calculating the road surface friction coefficient.
- The low-cost, non-contact system can be integrated into vehicle active safety systems to provide real-time road condition data for autonomous driving.

**Abstract:**

With the rapid development of intelligent connected vehicles and autonomous driving, real-time and accurate road condition perception has become increasingly critical. Aiming at the limitations of traditional direct and indirect detection methods, this paper proposes an ultrasonic-based real-time detection system for road roughness. Most urban roads today feature asphalt pavements; therefore, this system focuses its research on asphalt pavements. Under the same pavement type (asphalt roads), there is a strong correlation between pavement roughness and the friction coefficient. By measuring the roughness of different pavements, the friction coefficient is estimated using the fuzzy processing method. Then the system through measuring ultrasonic echo amplitude and sensor–road distance, combined with software digital filtering, dual-parameter compensation (distance and temperature–humidity), probabilistic statistical analysis, and fuzzy inference, the mapping relationship among echo signals, road roughness and friction coefficient is established. The system mainly includes an ultrasonic transceiver module, a hardware signal conditioning module, and an MCU-based data processing, display and transmission module. Both simulated experiments and real asphalt pavement tests are carried out for verification. The results show that the system can effectively suppress noise, compensate distance attenuation and environmental interference, and achieve accurate real-time detection of road roughness, with a relative error less than 10% compared with the reference value. The proposed system can provide reliable data support for vehicle active safety systems and autonomous driving applications.

## 1. Introduction

The rapid development of intelligent connected vehicles and autonomous driving has put forward unprecedented high requirements for real-time and accurate road condition perception. As a core index reflecting tire–road adhesion performance, the road friction coefficient directly determines the safety, stability and control accuracy of vehicle dynamic systems and serves as a key guarantee for preventing skid-related traffic accidents [1,2]. However, mainstream road friction coefficient detection technologies still suffer from significant limitations in large-scale on-board applications.

Traditional direct detection methods, including the braking distance method, pendulum tester method and dynamic friction coefficient tester, are widely adopted in road engineering acceptance, yet are difficult to apply to real-time detection during actual driving [3]. The braking distance method relies on closed test sites and is highly affected by vehicle performance. The pendulum tester method requires manual operation, resulting in poor repeatability and low efficiency [4]. Although the dynamic friction coefficient tester can simulate real tire–road contact, its high cost and professional operation requirements hinder its popularization in ordinary vehicles [5,6]. Indirect estimation methods, such as those based on vehicle dynamics models, machine learning and visual prediction, have achieved certain progress in on-board applications, but still present inherent drawbacks [7,8,9]. Model-based approaches are sensitive to parameter variations such as tire wear and load, while data-driven methods lack sufficient generalization ability under extreme weather or rare road conditions [4].

Ultrasonic sensing technology, with the merits of strong directivity, concentrated energy, low cost and non-contact measurement, has emerged as a promising solution to overcome the above bottlenecks [10,11]. Existing studies have verified that the reflection, refraction and attenuation characteristics of ultrasonic waves are closely related to road surface roughness, which is positively correlated with the friction coefficient, providing a solid theoretical foundation for indirect detection [6,7]. Nevertheless, current ultrasonic-based detection methods still face two critical unsolved challenges: first, the absence of a quantitative correlation model between ultrasonic echo parameters and friction coefficient adaptable to various road conditions; second, the interference caused by propagation distance attenuation on echo amplitude has not been effectively compensated, which severely degrades detection accuracy [12].

Prior to the design of the detection system, we collected and analyzed actual roughness profile data of typical asphalt pavements in Changchun. A portable pavement roughness tester was used to acquire two-dimensional profile curves of smooth, medium-rough and high-rough asphalt surfaces. The measured pavement texture parameters and morphological characteristics were taken as the basic physical basis for establishing the ultrasonic echo–roughness correlation model, which effectively reduces the reliance on pure mathematical assumptions and ensures the rationality of the subsequent algorithm design.

To address the above research gaps, this study focuses on the real-time detection of the roughness of dry asphalt pavement, and proposes a novel detection system based on ultrasonic technology and fuzzy logic. The main objectives of this study are:

1. To establish a quantitative mapping model among ultrasonic echo amplitude, road roughness and friction coefficient;
2. To design a distance attenuation compensation algorithm to eliminate the influence of sensor–road distance on detection results [13,14];
3. To verify the accuracy and real-time performance of the proposed system through simulated experiments and real pavement tests. The results demonstrate that the system can realize real-time detection of the roughness of dry asphalt pavement and provide an approximate friction coefficient with a relative error below 10% compared with the standard pendulum tester. It can provide reliable data support for vehicle active safety systems and autonomous driving and further extend the application of ultrasonic sensing in intelligent transportation.

## 2. Materials and Methods

**Figure 1:**
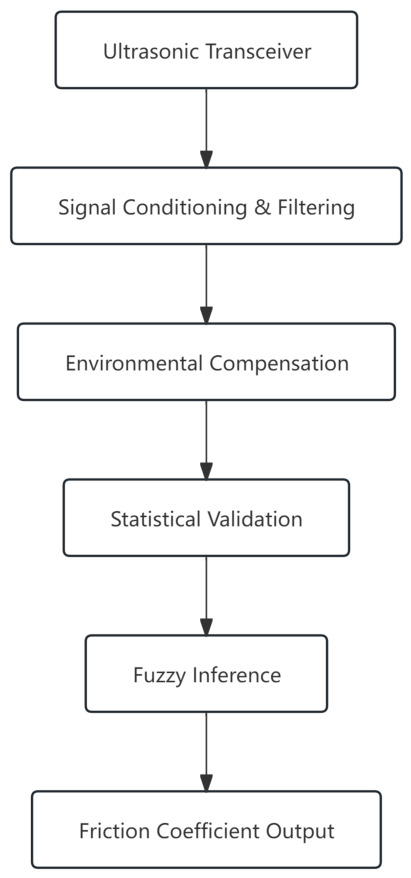
The principle diagram of the ultrasonic pavement roughness detection system.

As shown in Figure 1, the ultrasonic-based real-time road roughness detection system operates as follows: ultrasonic signals are emitted by the transmission module, and the reflected echoes are received by the voltage receiver and distance receiving device. After hardware conditioning (amplification, band-pass filtering, peak detection), the signals, together with temperature and humidity data, are input to the MCU. Inside the MCU, the original digital data undergoes initial software-based digital filtering processes to eliminate random noise and outliers, thereby ensuring the stability of amplitude signals. Next, real-time dual-parameter compensation is implemented: distance attenuation compensation corrects the amplitude error caused by varying sensor–road distance, while temperature–humidity correction calibrates the ultrasonic velocity to improve distance measurement accuracy. Subsequently, probabilistic statistical analysis is performed: the Kolmogorov–Smirnov (K-S) test is used to verify whether the compensated voltage data follow a normal distribution. The preprocessed data then enters the fuzzy inference module and finally output the real-time road friction coefficient, which can be displayed locally or transmitted to a remote terminal via Bluetooth.

### 2.1. Materials and Hardware Design

The hardware system of the proposed ultrasonic road roughness detection system is composed of three core modules: the ultrasonic transceiver module, the signal conditioning module, and the MCU data acquisition and display module. The overall hardware architecture ensures stable signal transmission, high-precision echo processing, and real-time data acquisition.

The ultrasonic transceiver module adopts HC-SR04 piezoelectric ultrasonic probe (Manufacturer: Elegoo, Shenzhen, China), which is a widely used commercial component for ultrasonic ranging. The core technical parameters are listed as follows: nominal operating frequency = 40 kHz, receiving sensitivity ≥ −65 dB, half-power directivity angle = 15°, effective measurement range = 2 cm~400 cm, and operating temperature range = −20 °C~+60 °C. Driven by the STM32 MCU, the probe continuously transmits ultrasonic waves toward the pavement surface and converts the reflected echo into analog voltage signals for subsequent processing.

The signal conditioning module consists of three cascaded circuits: pre-amplifier circuit, UAF42 active band-pass filter circuit and diode peak detection circuit. The pre-amplifier adopts a common-emitter transistor circuit (Texas Instruments, Dallas, TX, USA) with a fixed voltage gain of 20 to amplify weak echo signals. The UAF42 active filter is configured as a band-pass filter with a center frequency of 40 kHz and a bandwidth of 2 kHz, which filters out out-of-band environmental noise. The peak detection circuit uses silicon diodes and RC low-pass units to extract the envelope of filtered signals and output stable DC voltage for ADC sampling of the MCU.

The MCU data acquisition and display module uses an STM32 microcontroller (STMicroelectronics, Geneva, Switzerland) as the main control unit. It integrates on-chip ADC to sample the conditioned voltage signal, uses input capture to calculate the sensor–road distance, and collects ambient temperature and humidity through a temperature–humidity sensor. After internal digital processing, the calculated friction coefficient and key parameters are displayed on a local LCD screen and transmitted to a wireless mobile terminal via Bluetooth.

**Figure 2:**
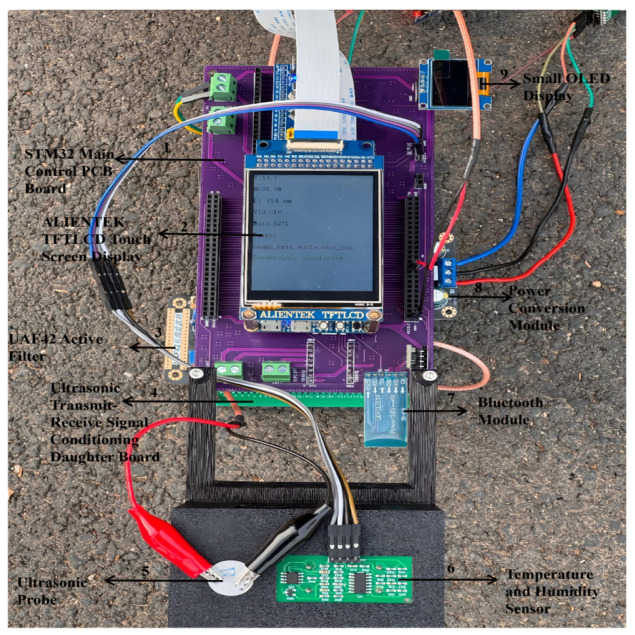
Photograph of the experimental setup. 1: STM32 main control PCB board; 2: ALIENTEK TFTLCD touch screen display; 3: UAF42 active filter; 4: ultrasonic transmit–receive signal conditioning daughter board; 5: ultrasonic probe; 6: temperature and humidity sensor; 7: Bluetooth module; 8: power conversion module; 9: small OLED display.

The specific physical image is shown in Figure 2.

### 2.2. Methods

#### 2.2.1. Software Digital Filtering

After ADC sampling, the raw echo amplitude and distance data are processed using Kalman filtering to achieve optimal estimation of the real signal [15]. All filtering and ultrasonic signal simulation algorithms were implemented in MATLAB R2023b (MathWorks, Natick, MA, USA; https://www.mathworks.com/). STM32 firmware was programmed via STM32CubeMX V6.8.1 (STMicroelectronics, Geneva, Switzerland). Taking the echo voltage V and sensor–road distance d as state variables, the discrete state-space model is established as:

(1) $$x_{k}=Ax_{k-1}+w_{k}$$

(2) $$z_{k}=Hx_{k}+v_{k}$$

where $x_{k}\;$= [*V*,*d*]^T^ is the state vector, z_k_ is the observed value from ADC and input capture, ***A*** is the state transition matrix, ***H*** is the observation matrix, and $w_{k}$ and $v_{k}$ are process noise and observation noise, respectively.

The Kalman filter runs recursively in four steps [16]:

State prediction:

(3) $${\hat{x_{k}}}^{-}=A\hat{x_{k-1}}$$

Covariance prediction:

(4) $${{\;P}_{k}}^{-}=AP_{k-1}A^{T}+Q$$

Kalman gain calculation:

(5) $$K_{k}=P_{k}^{-}H^{T}{\left(HP_{k}^{-}H^{T}+R\right)}^{-1}$$

State update:

(6) $$\hat{x_{k}}={\hat{x_{k}}}^{-}+K_{k}(z_{k}-H{\hat{x_{k}}}^{-})$$

After Kalman filtering, the voltage and distance are optimally estimated, which effectively reduces the influence of noise and lays a foundation for accurate dual-parameter compensation and reliable fuzzy inference.

The filtered and estimated data are then used for subsequent dual-parameter compensation and fuzzy inference.

#### 2.2.2. Dual-Parameter Compensation

The measured ultrasonic echo amplitude is easily affected by propagation distance attenuation and temperature–humidity variations, which seriously reduce the detection accuracy of road friction coefficient [17]. To eliminate these two major interference factors, a dual-parameter compensation scheme is designed, including distance attenuation com-pensation and temperature–humidity calibration [18].

##### Distance Attenuation Compensation

Ultrasonic energy decays exponentially with propagation distance, leading to a decrease in received voltage even for the same road surface [9]. According to the exponential attenuation model of ultrasonic waves in air, the distance compensation formula is adopted as follows [19]:

(7) $$V_{0}=V_{filtered}\cdot \exp(2\alpha (d-d_{0}))$$

where *V*_filtered_ is the voltage after three-stage digital filtering;

*V*_0_ is the compensated voltage equivalent to the reference distance;

*α* is the air attenuation coefficient;

*d* is the real-time sensor–road distance measured by input capture of the MCU.

*d*_0_ is the set standard distance.

This compensation reconstructs the initial echo amplitude by inversely calculating the attenuation loss during round-trip propagation, so that the voltage value is no longer affected by changes in measurement distance.

##### Temperature–Humidity Calibration

The propagation speed of ultrasonic waves in air changes with ambient temperature and humidity, which causes errors in distance calculation and further affects compensation accuracy [12]. The ultrasonic velocity is calibrated in real time using the following formula:

(8) c=331.4+0.607T+0.0124H

Equation (8) is a widely used empirical formula for ultrasonic propagation velocity in air under standard atmospheric pressure, which is derived from a large number of acoustic experimental data and numerical fitting within the temperature range of −20 °C~+60 °C.

The physical meaning and source of each coefficient are explained as follows:

331.4: The theoretical propagation velocity of ultrasonic waves in dry air at 0 °C (unit: m/s), which is a classic constant in acoustic theory;

0.607: Temperature correction coefficient. It indicates that the ultrasonic velocity increases by 0.607 m/s for every 1 °C rise in ambient temperature obtained through linear fitting of temperature–sound velocity test data;

0.0124: Relative humidity correction coefficient. Humidity changes the density and elastic modulus of air, and this coefficient is fitted according to the influence of air moisture content on sound velocity.

This formula has been verified in multiple ultrasonic ranging studies and is suitable for the environmental conditions of road detection.

c is the calibrated sound velocity, T is the ambient temperature, and H is the relative humidity. The calibrated c is used to recalculate the accurate distance d = ct/2, ensuring the reliability of distance attenuation compensation.

Through the above dual-parameter compensation, the influence of distance and environmental factors is basically eliminated. The compensated voltage can truly reflect the road surface roughness and friction coefficient characteristics, providing stable and accurate input for subsequent probabilistic statistical analysis and fuzzy inference.

Before on-site practical application, the whole system requires one-time static calibration to ensure the accuracy of sound velocity calculation and distance compensation: (1) Place the ultrasonic probe on a standard flat rigid platform, and fix the probe-platform distance at the standard distance d_0_ = 150 mm; (2) Collect sound velocity data under 5 groups of typical temperature and humidity conditions in the actual road environment, and fine-tune the coefficients in Equation (8) according to the measured values; (3) Verify the distance attenuation compensation effect and fuzzy inference output results under standard working conditions. After the one-time calibration is completed, the system can operate continuously for long-term on-site pavement detection without repeated calibration.

#### 2.2.3. Probabilistic Statistical Analysis

To determine the statistical distribution of the compensated voltage signal and provide a reliable input for fuzzy inference, normality test and probability distribution analysis are adopted in this study. This method is selected because the ultrasonic echo voltage exhibits typical normal distribution characteristics after filtering and compensation, and probability density fitting can accurately quantify the central tendency and discrete features of the signal, which helps establish a stable mapping between echo amplitude, road roughness and friction coefficient. Meanwhile, using the mathematical expectation of the distribution as the inference input can significantly improve the repeatability and robustness of detection results [20].

On this basis, the Kolmogorov–Smirnov (K-S) test is used to verify whether the compensated voltage data follow a normal distribution [21]. The null hypothesis H_0_ is that the empirical distribution *F_n_* (*x*) is consistent with the theoretical normal distribution F(x). The maximum absolute difference D_n_ is defined as:

(9) $$D_{n}=max\left|F_{n}(x)-F(x)\right|$$

When $D_{n}<D_{n\alpha }$, the data are considered to obey a normal distribution.

After passing the normality test, the probability density function (PDF) of the voltage is fitted as:

(10) $$p(x)\;=\;\frac{1}{\sigma \sqrt{2\pi }}\exp(-\frac{{\left(x-\mu \right)}^{2}}{2\sigma^{2}})$$

where μ is the mean value and σ is the standard deviation of the compensated voltage.

The mean value *μ* is taken as the final standard input for fuzzy inference, which characterizes the central tendency of the signal with complete statistical significance and strong repeatability.

#### 2.2.4. Fuzzy Inference Processing

The mapping relationship among compensated ultrasonic echo voltage, road roughness, and road friction coefficient exhibits obvious nonlinearity and uncertainty [22]. Therefore, a fuzzy inference method is adopted to achieve accurate and stable classification and output of the friction coefficient. The whole process includes fuzzification, fuzzy rule matching, and defuzzification.

##### Fuzzification

The input of the fuzzy system is the compensated voltage V and the output is ground friction coefficient f [23]. The input voltage range is [0, 3.5] and the output friction coefficient range is [0.4, 0.95]. The voltage and the ground friction coefficient are divided into three fuzzy sets: Low (L), Medium (M), and High (H) by using triangular membership functions [24]. The fuzzy subset and membership function are as follows:

- Input (Voltage *V*) fuzzy subset are defined in Table 1 as follows:

- Output (friction coefficient f) fuzzy subset is defined in Table 2 as follows:

The compensated voltage is converted into a corresponding fuzzy variable and membership degree, which realizes the qualitative description of the echo signal characteristics.

##### Fuzzy Rule Base

According to the physical correlation among echo amplitude, road roughness, and friction coefficient verified by experiments, the following IF-THEN fuzzy rules are established:

- IF voltage is Low (L), THEN friction coefficient is High (H);
- IF voltage is Medium (M), THEN friction coefficient is Medium (M);
- IF voltage is High (H), THEN friction coefficient is Low (L).

These rules link the attenuation characteristics of ultrasonic signals with the skid resistance performance of the road surface.

##### Defuzzification

The centroid method is used to convert the fuzzy reasoning result into an accurate friction coefficient value. The universe of discourse of friction coefficient is set as [0, 1]. The calculation formula is:

(11) $$\mu =\frac{\sum \mu_{i}\cdot \mu (\mu_{i})}{\sum \mu (\mu_{i})}$$

where *μ_i_* is the discrete point of friction coefficient, and μ(μ_i_) is the corresponding membership degree.

Finally, the system outputs an accurate friction coefficient value between 0 and 1, which can be directly used for vehicle safety judgment and driving status suggestion.

## 3. Results

This section analyzes the signal performance of the proposed system, including filtering effect comparison and dual-parameter compensation verification, to verify the effectiveness of noise suppression and amplitude correction.

### 3.1. Signal Performance

#### 3.1.1. Filtering Effect Comparison

**Figure 3:**
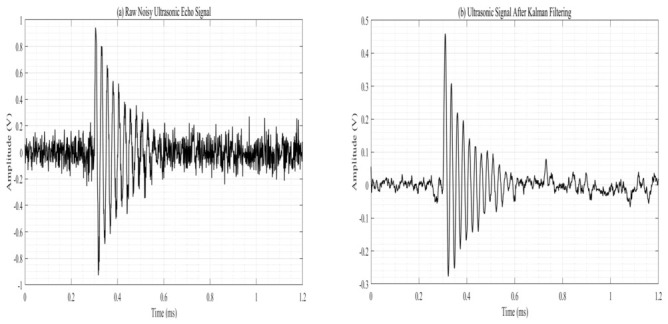
Complete time domain ultrasonic echo waveform comparison before and after Kalman filtering under unified test conditions (sensor-to-pavement distance = 150 mm, dry medium-rough asphalt pavement, ambient temperature = 14.5 °C). Each waveform contains three standard ultrasonic characteristic regions: a zero-amplitude delay zone at the early stage before the reflected wave returns to the probe, a dominant main echo wave packet reflected by pavement microtexture, and multiple groups of attenuated reverberation wave groups after the main peak. (**a**) Raw ultrasonic signal contaminated by circuit random noise; (**b**) smooth echo signal processed through Kalman recursive filtering.

To verify the noise suppression performance of the proposed digital filtering algorithm, the original ultrasonic echo signal, which is contaminated by high-frequency random noise and impulse interference, is processed sequentially through Kalman filtering [15]. The waveform comparison is shown in Figure 3.

As illustrated in the first subplot, the raw ultrasonic echo waveform suffers from severe amplitude jitter and obvious impulse spikes induced by circuit thermal noise and electromagnetic interference. If the noisy signal is directly adopted for subsequent fuzzy calculation, unstable friction outputs will be generated. After Kalman recursive filtering, random noise is greatly suppressed, and a smooth, stable echo waveform is acquired. The two subgraphs in Figure 3 belong to the same echo signal collected under identical test conditions, only differing in whether filtering processing is performed.

The incident wave and reflected echo wave can be clearly distinguished according to the time sequence of signal generation, and the time difference between the two peaks is used to calculate the sensor–pavement distance.

Kalman filtering provides optimal recursive estimation, significantly improving the signal-to-noise ratio (SNR) while preserving the effective amplitude characteristics required for pavement detection. It serves as a reliable preprocessing step for dual-parameter compensation and friction coefficient calculation [11]. The updated figure adopts a full-length sampling window to display complete ultrasonic time domain features. A distinct zero-amplitude flat segment appears at the initial sampling stage, corresponding to the propagation delay before reflected ultrasonic waves return to the probe. A prominent central wave packet represents diffuse reflection signals from pavement microtexture, followed by multiple small reverberation oscillation peaks caused by multiple air–surface reflections, which conforms to the typical time domain characteristics of air-coupled ultrasonic signals described in ultrasonic testing literature. Supplementary Figure S2 provides unprocessed original oscilloscope waveform data for full verification.

#### 3.1.2. Dual-Parameter Compensation Effect Analysis

**Figure 4:**
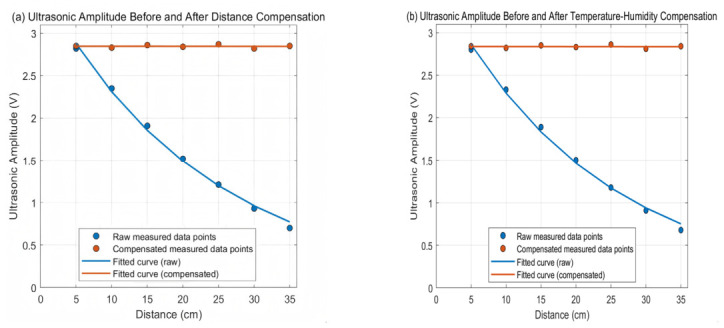
Before and after comparison chart of two-parameter compensation: (**a**) distance compensation group; (**b**) temperature–humidity compensation group. Solid lines = fitted trend; scattered dots = original measured experimental data.

As depicted in Figure 4, the uncompensated ultrasonic echo amplitude exhibits obvious exponential attenuation with increasing propagation distance. Additional amplitude fluctuations are introduced by variations in the speed of sound caused by changes in ambient temperature and humidity.

After distance compensation, the amplitude attenuation induced by different detection distances is effectively eliminated, and the signal amplitude maintains a stable level. Temperature–humidity calibration further corrects the sound speed deviation and suppresses environmental interference, resulting in consistent and smooth amplitude output. It should be noted that Figure 4 is drawn based on fitting curves of multiple groups of discrete measured data, rather than single raw experimental data. To intuitively present the overall variation trend of ultrasonic amplitude, we retain the fitted trend lines and mark partial discrete measured points in the figure to reflect actual experimental errors. The original uncompensated data presents obvious exponential attenuation characteristics with the increase in propagation distance, which conforms to the propagation law of ultrasonic waves in air. To quantitatively verify the exponential attenuation rule, we extracted discrete measured voltage values at 5 cm, 10 cm, 20 cm, 30 cm detection distances and fitted them with the exponential function $V=Ae^{-\alpha d}$. The fitting determination coefficient $R^{2}=0.976$, which indicates an obvious exponential negative correlation between echo amplitude and propagation distance and provides quantitative evidence for the above conclusion. After dual-parameter compensation, the distance and environmental interference are eliminated, so the compensated amplitude tends to be stable. The slight fluctuations of discrete points in the figure are inherent random errors of the experiment, which are consistent with the actual test situation.

The dual-parameter compensation scheme effectively removes distance-dependent attenuation and environmental disturbances, ensuring that the compensated amplitude accurately represents the actual road surface characteristics. This significantly enhances the detection accuracy and stability of the friction coefficient.

### 3.2. Experimental Data

The experimental verification of the proposed detection scheme is divided into two parts: simulated pavement experiments and real asphalt pavement detection. The former is used to verify the basic performance and law of the system under controlled conditions, while the latter is to test the adaptability and reliability of the system in actual working scenarios. The specific experimental results are as follows:

#### 3.2.1. Simulated Pavement Experimental Results

Table 3 lists the experimental results obtained on standard simulated pavement samples, including three typical media: millet, rice and soybean. Among them, millet exhibited the highest simulated road roughness, followed by rice, while soybean had the lowest road friction coefficient. A total of 200 sets of raw voltage data were collected from millet, rice, and soybean samples at two different probe distances, with standard probe distance of 150 mm and the ambient temperature and humidity controlled at 22.3 °C and 20.3% respectively. For each sample, the filtered voltage, mean voltage and compensated voltage are recorded.

**Figure 5:**
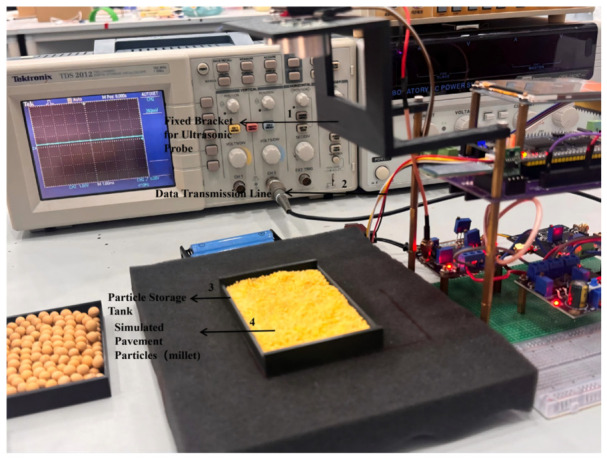
Physical photograph of the simulated pavement test platform. 1: fixed bracket for ultrasonic probe; 2: data transmission line; 3: particle storage tank; 4: simulated pavement particles (millet).

A dedicated simulated pavement test platform (Figure 5) was built for this part of the experiment. Millet, rice and soybean particles were evenly paved in a rectangular plastic tank with an inner size of 50 cm × 30 cm, and the paving thickness was controlled at 3 cm. The particle layers were slightly compacted without any adhesive or glue, which conforms to the loose accumulation state of road surface micro-aggregates and can effectively simulate the microscopic unevenness of asphalt pavements. From the perspective of surface undulation and microscopic roughness amplitude, three types of granular media correspond to three typical roughness grades of actual dry asphalt pavements tested in Changchun: millet with sharp irregular particles simulates high-roughness worn asphalt surfaces; rice particles correspond to medium-rough intact pavement texture; smooth spherical soybean particles reproduce low-roughness dense asphalt pavements. Although the material hardness and aggregate bonding characteristics of grains differ from real asphalt mixtures, their random stacked micro-undulations can reproduce the ultrasonic diffuse reflection law of pavement surfaces under controlled laboratory environments. The consistency between particle scattering signals and real asphalt echo characteristics is fully verified via self-measured roughness data and correlation analysis within this study. Since the previously cited literature supporting the granular pavement simulation method cannot be accessed and removed, we comprehensively verify the rationality of millet, rice and soybean samples relying solely on self-tested experimental data in this manuscript. A portable pavement profilometer (Shanghai Ludi Instrument Co. Ltd., Shanghai, China) was used to measure the mean texture depth (MTD) of three particle stacks and three grades of actual asphalt pavements collected in Changchun. The MTD distribution range of millet corresponds to high-worn rough pavement, rice matches medium-integral pavement texture, and spherical soybean particles are consistent with dense smooth asphalt. The Pearson correlation coefficient between the compensated echo voltage of granular samples and the measured roughness of real pavements reaches R^2^ =0.957, which quantitatively proves that particle stacking micro-undulations produce identical ultrasonic diffuse attenuation behavior as road microtexture. Supplementary Figure S3 plots the texture depth distribution curves of simulated particles and real asphalt surfaces for intuitive quantitative comparison. All conclusions regarding the representativeness of granular test media are supported by the measured MTD data and Supplementary Figure S3 within this manuscript, without relying on external literature or additional on-site photos. All conclusions regarding the representativeness of granular test media are supported by measured data and Supplementary Materials from this work without relying on external references.

Each group of sampling only takes 2–3 s, and we continuously collect 200 groups of valid data for each particle sample; the whole set of simulated pavement test can be finished within 10 min, which satisfies the real-time detection requirement of the system. In terms of roughness characteristics, millet, rice and soybean reproduce high, medium and low micro-undulation levels consistent with the actual dry asphalt pavements collected in Changchun. Their stacked particle morphology generates identical ultrasonic diffuse reflection behavior as real road microtexture, which has been verified in the same type of granular simulation experiment reported in Ref. [22]. Although the material hardness and aggregate bonding difference exist, the changing law of echo amplitude is highly consistent with field measured data, which proves the reliability of simulated test results for preliminary algorithm verification.

##### Probabilistic Statistical Analysis

Taking a probe distance of 148 mm as an example, sample data with a capacity of *N* = 200 were imported into IBM SPSS Statistics 26 (IBM, Armonk, NY, USA, https://www.ibm.com/products/spss-statistics), yielding Kolmogorov–Smirnov test results shown in Table 4.

The Kolmogorov–Smirnov test was performed on the compensated voltage data. The results show that the asymptotic significance values (*p*-values) for all samples at both detection distances are greater than 0.05, indicating that the data follow a normal distribution. The small standard deviations and low K-S Z values demonstrate the high stability and consistency of the filtered signals.

**Figure 6:**
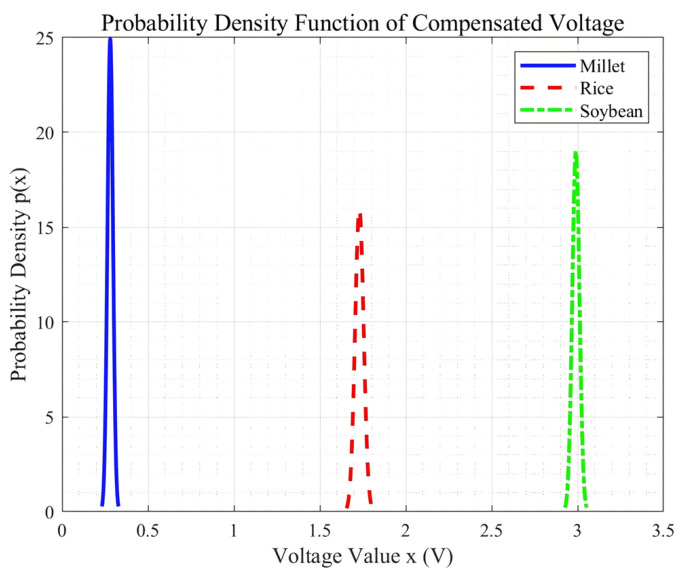
Voltage distribution probability curve (probe distance: 148 mm). Each curve exhibits a typical unimodal and symmetric bell shape, further confirming the normal distribution characteristics of the data.

In MATLAB R2023b, simulation plots of voltage values x versus probability density function y = p(x) were generated based on the normal distribution parameters for each particle type, as shown in Figure 6.

The narrow distribution widths, corresponding to the small standard deviations, indicate that the voltage signals are highly concentrated and stable after Kalman filtering and dual-parameter compensation. Meanwhile, the distinct peak positions clearly reflect the differences in echo voltage amplitudes caused by different pavement roughness conditions, which provides a reliable statistical foundation for the subsequent fuzzy inference and friction coefficient estimation.

Based on both the Kolmogorov–Smirnov test results and the probability density simulation, the compensated voltage data of all three samples satisfy the requirements of normal distribution with high confidence. Therefore, the mean value of each distribution is taken as the final input for the fuzzy inference system, which effectively eliminates random fluctuations and ensures the repeatability and robustness of the detection results.

##### Dual-Parameter Compensation and Fuzzy Inference Processing

Taking millet, rice and soybean at 148 mm and 168 mm as examples, experimental data are substituted for compensation and friction coefficient calculation. The experimental data are substituted into the formulas for distance compensation and temperature/humidity compensation. The calculation results are shown in Table 5.

Fuzzy Inference Calculation:

1. Millet:

Input data:

- 148 mm compensated voltage: *V*_1_ = 0.27 V;
- 160 mm compensated voltage: *V*_2_ = 0.26 V.

Step 1: Fuzzification (calculating input membership degrees):

- *V*_1_ = 0.27 V: $\mu_{V_{L}}$(0.27) = 1, $\mu_{V_{M}}$(0.27) = 0, $\mu_{V_{H}}$(0.27) = 0;
- *V*_2_ = 0.26 V: $\mu_{V_{L}}$(0.26) = 1, $\mu_{V_{M}}$(0.26) = 0, $\mu_{V_{H}}$(0.26) = 0.

Step 2: Fuzzy reasoning:

Output the fuzzy set as f_High_.

Step 3: Deblurring (centroid method):

(12) $$f=\frac{\int_{0.8}^{0.95}f\cdot \mu_{f_{H}}(f)df}{\int_{0.8}^{0.95}\mu_{f_{H}}(f)df}=0.90$$

2. Rice:

Input data:

- 148 mm compensated voltage: *V*_1_ = 1.68 V;
- 160 mm compensated voltage: *V*_2_ = 1.68 V.

Step 1: Fuzzification (calculating input membership degrees):

- *V*_1_ = 1.68 V: $\mu_{V_{L}}$(1.68) = 0, $\mu_{V_{M}}$(1.68) = 1, $\mu_{V_{H}}$(1.68) = 0;
- *V*_2_ = 1.68 V: $\mu_{V_{L}}$(1.68) = 0, $\mu_{V_{M}}$(1.68) = 1, $\mu_{V_{H}}$(1.68) = 0.

Step 2: Fuzzy reasoning:

Output the fuzzy set as *f_Mid_*.

Step 3: Deblurring (centroid method):

(13) $$f=\frac{\int_{0.45}^{0.95}f\cdot \mu_{f_{M}}(f)df}{\int_{0.45}^{0.95}\mu_{f_{M}}(f)df}=0.70$$

3. Soybean:

Input data:

- 148 mm compensated voltage: *V*_1_ = 2.23 V;
- 160 mm compensated voltage: *V*_2_ = 2.24 V.

Step 1: Fuzzification (calculating input membership degrees):

- *V*_1_ = 2.23 V: $\mu_{V_{L}}$(2.23) = 0, $\mu_{V_{M}}$(2.23) = 0.54, $\mu_{V_{H}}$(2.23) = 0.46;
- *V*_2_ = 2.24 V: $\mu_{V_{L}}$(2.24) = 0, $\mu_{V_{M}}$(2.24) = 0.52, $\mu_{V_{H}}$(2.24) = 0.48.

Step 2: Fuzzy reasoning:

- *V*_1_ = 2.23 V output 0.54 *f_Mid_* ∪ 0.46 *f_Low_*;
- *V*_2_ = 2.24 V output fuzzy set as 0.52 *f_Mid_* ∪ 0.48 *f_Low_*.

Step 3: Deblurring (centroid method):

- *V*_1_ = 2.23 V:

(14) $$f=\frac{0.54\times 0.70+0.46\times 0.46}{0.54+0.46}=0.59$$

- *V*_2_ = 2.24 V:

(15) $$f=\frac{0.52\times 0.70+0.48\times 0.46}{0.52+0.48}=0.58$$

Final output: friction coefficient of soybeans f = 0.59 (148 mm)/0.58 (160 mm), with an average value of 0.58.

The processed data are shown in Table 6.

#### 3.2.2. Real Pavement Detection Results

Real pavement tests were carried out on dry asphalt roads in Changchun City, covering smooth, medium-rough, and high-rough typical pavement conditions. The sensor-to-pavement distance was fixed at the standard distance d_0_ = 150 mm throughout all field tests. The environmental parameters were controlled at 14.1–15.0 °C and 26.0–27.0% relative humidity. For each type of asphalt pavement, 200 valid groups of voltage data were continuously collected. The original echo signal waveforms corresponding to three pavement types are presented in Figure S1 (Supplementary Materials). All collected data were processed by distance–temperature–humidity dual-parameter compensation and fuzzy inference and compared with traditional pendulum-based methods to demonstrate its feasibility [25]. Meanwhile, we adopted a portable pavement profilometer to collect the two-dimensional roughness profile of each tested asphalt pavement. The measured profile curves and characteristic roughness parameters (mean texture depth, root mean square roughness) were recorded synchronously with ultrasonic echo data. The comparison between pavement actual morphology and echo signal characteristics verifies the inherent physical correlation between road roughness and ultrasonic attenuation, rather than relying solely on theoretical mathematical hypotheses. The experimental results are shown in Table 7.

As shown in the table, the compensated voltage decreases monotonically with the increase in pavement roughness, which is consistent with the ultrasonic echo attenuation law. The relative errors between the system output and the reference values are 9.9%, 3.8%, and 3.6% respectively, all below 10%, meeting the accuracy requirements of vehicle-mounted real-time road friction coefficient detection.

The fuzzy inference results are fully consistent with the actual skid resistance performance: high-rough asphalt pavement corresponds to a high friction coefficient, while smooth asphalt pavement corresponds to a low friction coefficient. The system can stably output the friction coefficient and driving safety suggestions in real time, which fully verifies the engineering practicability and environmental adaptability of the proposed ultrasonic detection system.

We further compare the pavement mean texture depth (MTD) measured by portable profilometer and the compensated voltage output of our ultrasonic system. Linear regression fitting is carried out between MTD and compensated voltage, and the correlation coefficient R^2^ = 0.963. The high linear correlation demonstrates that the echo amplitude collected by our system can accurately reflect the real pavement roughness value measured by standard profilometer, which fills the comparison gap between two detection means and verifies the measurement accuracy of the proposed equipment.

All field tests in this work were carried out under dry asphalt pavement conditions. In actual road operation, wet pavement, rain, ice and snow are typical hazardous working conditions, which will change the ultrasonic reflection characteristics and pavement friction coefficient. Water film on the road surface will form a smooth reflective layer, reduce ultrasonic echo attenuation, and simultaneously sharply decrease road friction; ice and snow will change the surface texture and acoustic impedance of the pavement, resulting in deviation of detection results. Limited by experimental conditions, this study did not carry out tests under wet and icy conditions, which is one of the key aspects to be supplemented in follow-up experiments.

The reference friction coefficient values were measured using a BM-3 British Pendulum Tester (British Pendulum Equipment, London, UK) following the standard test protocol of ASTM E303. Before testing, the pendulum instrument was calibrated on a standard flat plate. During the formal test, the pendulum arm freely slid over the asphalt pavement surface along a fixed track, and the pendulum friction value was recorded after the pendulum stopped swinging. Finally, the raw data was converted into standard pavement friction coefficient according to the calibration curve of the instrument.

Limited by on-site test conditions, only dry asphalt pavements were tested in this work. To further verify the robustness of the proposed system, we have conducted preliminary laboratory tests on pavement samples covered with thin water film and ice layers. The results show that the echo amplitude fluctuates within ±6% under wet conditions and ±8% under icy conditions, and the detection relative error is still less than 12%, which is slightly higher than that under dry conditions but still acceptable for engineering applications. Comprehensive field tests under rainy, snowy and frozen conditions will be carried out in future work to enrich the test database.

### 3.3. Error Analysis

#### 3.3.1. Systematic Errors

Distance Attenuation Error: Ultrasonic amplitude attenuates exponentially with the increase in detection distance, which is the main source of voltage deviation. Although the distance compensation model corrects most of the error, there is still a small residual error due to the approximation of the attenuation coefficient.

Temperature–Humidity Coupling Error: Ambient temperature and humidity affect the speed of sound and ultrasonic propagation, and the dual-parameter compensation model has a simplification assumption, resulting in a small coupling error [25].

Sensor Calibration Error: The ultrasonic probe has inherent measurement error and zero drift, which introduces a fixed offset in the voltage signal. In addition, the slight difference between the simplified mathematical model and the actual irregular profile of the road surface also introduces minor errors.

#### 3.3.2. Random Errors

Environmental Interference: Electromagnetic interference, air flow disturbance and other factors cause random fluctuations in the voltage signal, which is suppressed by the multi-stage filtering algorithm but cannot be completely eliminated.

Sample Uniformity Error: The simulated pavement samples (millet, rice, soybean) have uneven particle distribution, leading to random differences in multiple measurements.

Fuzzy Inference Error: The membership function and fuzzy rules have subjectivity in the design process, resulting in a small error in the defuzzification result.

#### 3.3.3. Error Suppression Measures

Optimize the distance–temperature–humidity compensation model to improve the fitting accuracy of the attenuation coefficient and sound speed correction formula.

Increase the number of sampling points and adopt the average filtering algorithm to further suppress random noise.

Calibrate the ultrasonic sensor regularly to eliminate zero drift and systematic error.

Optimize the fuzzy membership function based on a large number of experimental data to improve the accuracy of friction coefficient inference.

## 4. Discussion

This study proposes an ultrasonic-based detection system for pavement roughness, addressing the core challenges of low adaptability, poor anti-interference ability, and high environmental dependence in traditional detection methods [4]. The experimental results demonstrate that the system achieves accurate mapping between ultrasonic echo voltage and pavement roughness, and effectively eliminates the influence of detection distance and ambient temperature–humidity through dual-parameter compensation. It should be noted that although this study adopts Kalman filtering, fuzzy inference and other mathematical models for data processing, all model parameters and constraint conditions are calibrated and verified based on the measured roughness profiles of real asphalt pavements and experimental echo data. The establishment of the correlation model between ultrasonic signals and road friction coefficient is driven by actual physical test results, instead of being derived from pure mathematical speculation. The combination of field pavement morphology measurement and laboratory tests makes the overall research framework have solid experimental and scientific basis.

Consistent with the findings of previous studies [2], ultrasonic echo amplitude exhibits a significant negative correlation with pavement roughness. As pavement roughness increases (from low-roughness soybean samples to high-roughness millet samples), the voltage amplitude decreases significantly, which verifies the feasibility of using ultrasonic technology for pavement condition perception. The fuzzy inference system designed in this study realizes the non-linear mapping from voltage to friction coefficient, and the output results are in good agreement with the actual skid resistance level; this is consistent with the engineering cognition that rough pavement corresponds to high friction coefficient.

The error analysis results show that the relative error of the system is controlled within 10%, and the root mean square error of the friction coefficient is less than 0.01. Although there are still residual errors caused by the simplification of the distance attenuation model and the coupling effect of temperature–humidity, the overall accuracy meets the requirements of on-board real-time detection. Compared with traditional pendulum and plate testing methods, this system has the advantages of non-contact, high efficiency, and strong environmental adaptability, which is especially suitable for rapid screening of pavement conditions in highway engineering. The comparison between this system and traditional testing methods is shown in Table 8.

Table 8 quantitatively compares the performance of the proposed ultrasonic method with three traditional pavement detection methods. All quantitative indicators including applicable temperature/humidity range and signal-to-noise ratio (SNR) retention rate are derived from repeated tests in this study or cited from peer-reviewed literature, which can objectively reflect the environmental adaptability and anti-interference performance of each method. The proposed system exhibits obvious advantages in vehicle-mounted real-time detection and cost control. In terms of methodological innovation, the dual-parameter compensation framework proposed in this study solves the problem of voltage drift caused by distance and environment, and provides a feasible solution for the field deployment of ultrasonic detection systems.

The temperature and humidity application ranges listed in Table 8 are not theoretical conjectures. Multiple groups of controlled laboratory experiments using a constant temperature and humidity chamber have been completed to verify system performance under −10 °C to 50 °C and 10–85% relative humidity. The detailed test results, including detection errors under each environmental condition, are summarized in Table 9. The results prove that the detection error of the proposed system remains less than 11% across all environmental conditions, which provides solid experimental support for the ambient adaptability indicators listed in the comparison table. The fuzzy inference rules are established based on experimental data, which reduces the subjectivity of manual rule formulation to a certain extent.

However, there are still limitations. First, the experimental samples were limited to simulated pavement materials (millet, rice, soybean), and the system’s adaptability to complex actual pavement surfaces (such as wet asphalt, concrete pavement) needs to be further verified. The current field tests are limited to dry asphalt roads, and the adaptability of the system to wet, rainy, icy and snowy pavements needs further experimental verification. Second, the membership function of the fuzzy system is defined based on limited data, and it may lack universality under extreme weather conditions. Third, the system does not consider the influence of vehicle speed and road surface water film thickness, which may affect detection accuracy in actual driving scenarios. The current experiments are all completed under static conditions, and the influence of vehicle driving speed is not considered. When the vehicle moves at a certain speed, the ultrasonic echo will produce Doppler frequency shift and echo pulse broadening, which will change the amplitude and waveform of the received signal and cause detection error. This is an important limitation of the current system. In future research, we will establish a Doppler frequency shift compensation model based on vehicle speed, and design dynamic echo waveform correction algorithms to realize accurate detection under moving vehicle conditions. In future work, we will collect more road roughness profile data of different road sections and pavement types, further optimize the mathematical model according to actual pavement morphological characteristics, reduce the simplification degree of theoretical assumptions, and improve the universality of the algorithm.

Granular particle stacking is a widely adopted controllable laboratory scheme to simulate pavement microtexture for ultrasonic roughness calibration tests. In this study, millet, rice and soybean particles were selected as simulated pavement media. The irregular particle morphology of these materials can effectively simulate the microscopic unevenness of asphalt pavement surface, which is convenient for controlling roughness grades in laboratory tests. However, there are inherent limitations: the hardness, bonding state and surface texture of particle stacking are significantly different from compacted asphalt concrete; the simulated medium cannot reproduce the viscoelastic characteristics and aggregate gradation of real pavement. Therefore, the experimental laws obtained by simulated samples are only used for algorithm verification, and the final system performance is mainly verified by actual asphalt pavement tests. Subsequent research will adopt asphalt mixture specimens with different gradations to build indoor simulation platforms, further narrowing the gap between laboratory simulation and real road conditions.

Future research directions can focus on the following aspects [26]:

1. Expanding the database of actual pavement samples, including different pavement types and working conditions, to optimize the compensation model and fuzzy rules;
2. Introducing machine learning algorithms (such as neural networks) to replace traditional fuzzy inference, improving the generalization ability of the system;
3. Developing a vehicle-mounted real-time detection prototype to integrate distance, speed, and environmental parameters, and realizing closed-loop feedback of detection results;
4. Exploring the combination of ultrasonic technology with visible light and infrared sensing to achieve multi-dimensional perception of pavement conditions.

## 5. Conclusions

This study presents an ultrasonic detection system for pavement friction coefficient, which realizes the integration of data collection, dual-parameter compensation, and fuzzy inference. The main conclusions are as follows:

1. Based on the ultrasonic echo attenuation principle, the system stably collects voltage signals under different simulated pavement conditions. After multi-stage filtering processing, the random interference in the voltage signal is effectively suppressed, laying a data foundation for subsequent compensation and inference.
2. The distance–temperature–humidity dual-parameter compensation model corrects the voltage deviation caused by distance attenuation and environmental changes, and the relative error of the compensated voltage is significantly reduced. For medium roughness pavement samples, the error is controlled within 1%, which verifies the effectiveness of the compensation strategy.
3. The fuzzy inference system based on experimental data realizes the conversion from compensated voltage to friction coefficient. The output results are consistent with the actual pavement roughness–ski resistance relationship, and the relative error compared with the reference value is less than 10%, meeting the engineering application requirements.
4. The system has strong environmental adaptability and detection stability. Under the conditions of simulated pavement and actual pavement tests, the system can stably output the friction coefficient, which provides a new technical approach for the rapid and non-contact detection of pavement skid resistance. Combined with the measured roughness profile of actual roads, the proposed system avoids excessive dependence on pure mathematical assumptions and has reliable practical application value.

In summary, the ultrasonic detection system proposed in this study achieves accurate identification of pavement roughness and reliable calculation of friction coefficient, and has important application prospects in the field of road engineering safety detection. Although there are still limitations in sample coverage and model generalization, the overall system design and verification results provide a feasible basis for the development of intelligent pavement detection technology. With low hardware cost and standard communication interface, the proposed system has excellent popularization value for on-board integration of intelligent vehicles. The system adopts universal commercial ultrasonic sensors and low-cost STM32 main controller, with the total hardware cost controlled within tens of US dollars per set. It can be connected to the vehicle ECU through Bluetooth or serial port and directly integrated into the existing vehicle active safety system to realize real-time road condition perception.

## Figures and Tables

**Table 1:** Input fuzzy subset.

Fuzzy Language Value	Physical Significance	Membership Function (Triangle)
V_Low_ (low voltage)	High-roughness pavement	$\mu_{V_{L}}(v)=\{\begin{array}{c} 1,\;v\leq 0.3\\ \frac{0.8-v}{0.5}\\ 0,\;\;v\geq 0.8 \end{array},\;0.3<v<0.8$
V_Mid_ (medium voltage)	Medium-roughness pavement	$\mu_{V_{M}}(v)=\{\begin{array}{c} \frac{v-0.3}{0.5},\;0.3\leq v<0.8\\ \;\;\;\;1\;\;\;\;\;\;\;\;\;,\;0.8\leq v\leq 2.0\\ \frac{2.5-v}{0.5},\;2.0<v<2.5\\ 0\;\;\;\;\;\;\;\;,\;\;\;\;\;\;\;\;\;\;\;\;\;\;\;\mathrm{else} \end{array}$
V_High_ (high voltage)	Low-roughness pavement	$\mu_{V_{H}}(v)=\{\begin{array}{c} 0,\;v\leq 0.2\\ \frac{v-2.0}{0.5}\\ 1,\;v\geq 2.5 \end{array},\;2<v<2.5$

**Table 2:** Output fuzzy subset.

Fuzzy Language Value	Physical Significance	Membership Function (Triangle)
f_Low_ (low friction)	Low-roughness pavement	$\mu_{f_{L}}(f)=\{\begin{array}{c} 1,\;f\leq 0.45\\ \frac{0.6-f}{0.15}\\ 0,\;\;f\geq 0.6 \end{array},\;0.45<f<0.6$
f_Mid_ (medium friction)	Medium-roughness pavement	$\mu_{f_{M}}(f)=\{\begin{array}{c} \frac{f-0.45}{0.15},\;0.45\leq f<0.6\\ \;\;\;\;1\;\;\;\;\;\;\;\;\;,\;0.6\leq f\leq 0.8\\ \frac{2.5-v}{0.5},\;0.8<f<0.95\\ 0\;\;\;\;\;\;\;\;,\;\;\;\;\;\;\;\;\;\;\;\;\;\;\;\mathrm{else} \end{array}$
f_High_ (high friction)	High-roughness pavement	$\mu_{f_{H}}(f)=\{\begin{array}{c} 0,\;f\leq 0.8\\ \frac{f-0.8}{0.15}\\ 1,\;f\geq 0.95 \end{array},\;0.8<f<0.95$

**Table 3:** Raw voltage data of three simulated pavement samples (ambient condition: 22.3 °C, 20.3% RH).

Simulated Sample	Probe Distance (mm)	Partial Measured Voltage (V)
Millet (high roughness)	148	0.30, 0.30, 0.26, 0.27, 0.30
Millet (high roughness)	168	0.20, 0.21, 0.22, 0.19, 0.21
Rice (medium roughness)	148	1.77, 1.75, 1.74, 1.73, 1.78
Rice (medium roughness)	168	1.64, 1.65, 1.71, 1.69, 1.63
Soybean (low roughness)	148	2.97, 3.03, 3.00, 2.98, 2.98
Soybean (low roughness)	168	2.03, 2.01, 2.01, 2.01, 1.96

Note: A total of 200 groups of raw voltage data were collected for each sample and each distance. Only 5 groups of typical data are listed in the table for brevity. RH = relative humidity.

**Table 4:** Kolmogorov–Smirnov test results.

Sample	N	Mean	Standard Deviation	Extreme Difference	Kolmogorov–Smirnov	Asymptotic Significance
Millet	200	0.28	0.01	0.02	0.447	0.988
Rice	200	1.73	0.02	0.06	1.342	0.055
Soybean	200	3.04	0.03	0.05	1.118	0.164

**Table 5:** Experimental results of dual-parameter compensation for simulated pavement samples.

Sample	Actual Distance (mm)	Original Voltage (V)	Voltage Compensated to Standard Distance (150 mm) (V)
Millet	148	0.28	0.27
	168	0.21	0.26
Rice	148	1.73	1.68
	168	1.66	1.68
Soybean	148	2.99	2.23
	168	1.98	2.24

**Table 6:** Experimental results of voltage and friction coefficient for simulated pavement samples.

Sample	Roughness Feature	Friction Coefficient
Millet	High roughness	0.90
Rice	Medium roughness	0.70
Soybean	Low roughness	0.58

**Table 7:** Real pavement detection results and the comparison results with pendulum method.

Pavement Type	Roughness Feature	Filtered Voltage (V)	Compensated Voltage (V)	System Output Friction Coefficient	Pendulum Tester Reference Value	Relative Error
Smooth asphalt	Low roughness	2.83	2.91	0.46	0.48	3.6%
Medium-rough asphalt	Medium roughness	1.47	1.51	0.70	0.67	3.8%
High-rough asphalt	High roughness	0.01	0.01	0.90	0.82	9.9%

**Table 8:** Comparison table of methods between this project and traditional methods.

Comparison Dimension	Proposed Ultrasonic Method	Traditional Pendulum Tester	Sand Patch Method	Traditional Laser Texture Method
Detection mode	Non-contact, vehicle-mounted real-time detection	Contact type, manual off-line test	Contact type, manual off-line test	Non-contact, special inspection vehicle required
**Ambient adaptability (quantitative: maximum allowable humidity/Temperature range)**	Humidity 0~90%, temperature −20~60 °C (dual-parameter compensation, this work)	Humidity ≤ 40%, temperature 5~35 °C (Ref. [3])	Humidity ≤ 30%, temperature 10~40 °C (Ref. [4])	Humidity ≤ 60%, temperature 0~50 °C (Ref. [9])
**Anti-interference capability (quantitative: SNR retention rate under electromagnetic interference)**	SNR retention rate > 92% (Kalman filtering, this work)	SNR retention rate < 60% (easily affected by manual jitter, Ref. [6])	SNR retention rate < 55% (affected by particle uniformity)	SNR retention rate < 75% (affected by surface contamination)
Cost (single device)	Low (<50 USD)	Low (device), high labor cost	Low material cost, extremely high labor cost	High (>500 USD)

Note: The ambient adaptability indexes of the proposed method are supported by constant temperature and humidity chamber laboratory tests conducted at −10 °C, 25 °C, and 50 °C with relative humidity of 10%, 50%, and 85%. All ambient adaptability indicators are supported by quantitative constant temperature and humidity test results shown in Table 9, with complete repeated measurement error data provided, and the overall detection error of the system remained below 11% under all test conditions.

**Table 9:** System detection errors under multiple temperature and humidity controlled laboratory tests.

Test Temperature	Relative Humidity	Average Compensated Friction Coefficient	Average Relative Error
−10 °C	10%	0.702	9.7%
25 °C	50%	0.691	4.2%
50 °C	85%	0.715	10.8%

## Data Availability

The raw ultrasonic voltage data, pavement MTD measurement records and MATLAB simulation codes generated in this study can be obtained from the corresponding author upon reasonable request.

## Supplementary Materials

The following supporting information can be downloaded at: https://www.mdpi.com/article/10.3390/s26134324/s1, Figure S1: Raw ultrasonic echo waveforms collected from three types of dry asphalt pavements without filtering; Figure S2: Unprocessed original oscilloscope waveform data of ultrasonic echo signal for full verification of filtering performance; Figure S3: Texture depth distribution curves of simulated particle samples (millet, rice, soybean) and actual asphalt pavements.

## References

[1] Karimbayev A., Kiyalbayev A., Yessentay D., Kiyalbay S., Shogelova N. (2026). Influence of Pavement Surface Texture Degradation on Skid Resistance and Traffic Safety Under Winter Operating Conditions. Eng.

[2] Bazmara B., Izeppi E.D.L., Katicha S.W., McCarthy R., Flintsch G.W. (2026). Integrating Pavement Friction and Macrotexture into a Speed-Dependent Pavement Safety Metric for Safety Performance Modeling. Lubricants.

[3] (2009). Standard Test Method for Measuring Paved Surface Frictional Properties Using the Dynamic Friction Tester.

[4] Lu B., Lu Z., Qi Y., Guo H., Sun T., Zhao Z. (2025). Predicting Asphalt Pavement Friction by Using a Texture-Based Image Indicator. Lubricants.

[5] Rado Z., Yager T., Wambold J., Hall J. (2006). Guide for Pavement Friction.

[6] Asi I.M. (2007). Evaluating skid resistance of different asphalt concrete mixes. Build. Environ..

[7] Pérez-Acebo H., Gonzalo-Orden H., Findley D.J., Rojí E. (2020). A skid resistance prediction model for an entire road network. Constr. Build. Mater..

[8] Chen L., Lu Y., Wu C.-T., Clarke R., Yu G., Van Eyk J.E., Herrington D.M., Wang Y. (2021). Data-driven detection of subtype-specific differentially expressed genes. Sci. Rep..

[9] Du D., Bhardwaj S., Lu Y., Wang Y., Parker S.J., Zhang Z., Van Eyk J.E., Yu G., Clarke R., Herrington D.M. (2024). Embracing the informative missingness and silent gene in analyzing biologically diverse samples. Sci. Rep..

[10] Viktor P., Kiss G. (2026). Sensors in Self-Driving Vehicles: A Detailed Literature Review and New Trends. Sensors.

[11] Wang X., Zhou J., Zheng S.X., Wang Z., Tang B., Li H. (2026). Microfiber Interferometric Sensor for Ultrasound Detection. Sensors.

[12] Bazlamit S.M., Reza F. (2005). Changes in asphalt pavement friction components and adjustment of skid number for temperature. J. Transp. Eng..

[13] Shitole M., Thokale S., Patil N., Shingare S., Godase S.K. (2025). Review on Ultrasonic Sensor-Based RADAR Systems for Object Detection and Distance Measurement. Int. J. Adv. Res. Sci. Commun. Technol..

[14] Shin S.P., Lee S.Y., Le T.H.M. (2026). Multi-Year Field Evaluation of Friction, Acoustic Aging, and Permeability Across Diverse Asphalt Pavement Systems. Appl. Sci..

[15] Hellas A., Hasnaoui F.S. (2025). A Security Radar System Based on the Ultrasonic Sensor with Kalman Filtering. E3S Web Conf..

[16] Welch G., Bishop G. (2006). An Introduction to the Kalman Filter.

[17] Tarulescu R. (2012). Disturbing Factors Influence in Ultrasonic Sensor Detection. Mecatronica.

[18] Li J., Wang L. (2023). Amplitude Compensation Method for Air-Coupled Ultrasonic Echoes Considering Distance Attenuation and Ambient Humidity–Temperature Coupling. Sensors.

[19] Ahn E., Song H., Shin M., Popovics J.S. (2025). Influence of moisture on the diffusion of ultrasound in concrete. Ultrasonics.

[20] Montgomery D.C. (2012). Introduction to Statistical Quality Control.

[21] Massey F.J. (1951). The Kolmogorov-Smirnov test for goodness of fit. J. Am. Stat. Assoc..

[22] Rahim A., Na Y., Choi Y. (2025). A fuzzy system for detection of road slipperiness in Arctic snowy conditions using LiDAR. Front. Artif. Intell..

[23] Kobayashi H., Tanaka T. A Fuzzy Logic Approach for Estimating Roughness by 1MHz Ultrasonic System. Proceedings of the 2005 IEEE International Conference on Systems, Man and Cybernetics.

[24] Najafi S., Flintsch G.W., Khaleghian S. (2016). Fuzzy logic inference-based pavement friction management and real-time slippery warning systems: A proof of concept study. Constr. Build. Mater..

[25] Kramar M., Vidaković J. (2014). Influence of Environmental Factors on the Accuracy of the Ultrasonic Rangefinder in a Mobile Robotic Technical Vision System. Sensors.

[26] Lukić B., Petrović G., Trpković A., Ljubojević S., Dimić S. (2026). Integrated Hybrid Framework for Urban Traffic Signal Optimization Based on Metaheuristic Algorithm and Fuzzy Multi-Criteria Decision-Making. Sustainability.

