# Supplementary material for: Real-Time Detection System for Road Roughness Based on Ultrasonic Technology
Source: Sensors (Basel). 2026 Jul 7;26(13):4324. doi: 10.3390/s26134324 (PMC13364443; doi:10.3390/s26134324)
Supplement: Supplementary file 1 [file sensors-26-04324-s001.zip › sensors-4336228-supplementary.pdf]

Supporting Information

Figure Captions for Supplementary Figures

1. Figure S1

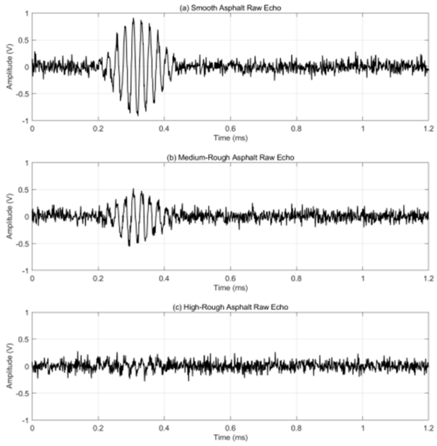

Figure S1. Raw noisy ultrasonic echo waveforms corresponding to smooth, medium-rough, and high-rough dry asphalt pavements collected at a fixed sensor-pavement distance of 150 mm. Obvious random circuit noise and impulse jitter exist in all three groups of unfiltered signals, which visually demonstrates the necessity of Kalman filtering for signal denoising in the proposed detection system.

2. Figure S2

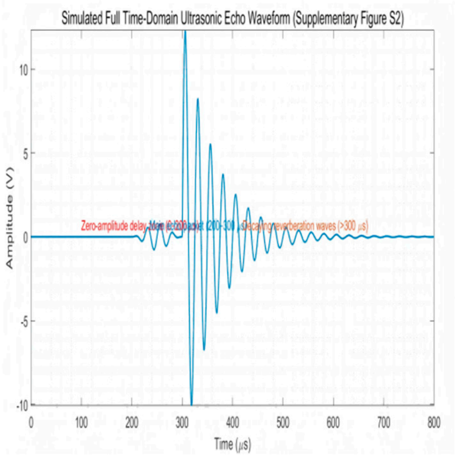

Figure S2. Unprocessed raw oscilloscope waveform of ultrasonic echo signals acquired under standard test conditions (150 mm detection distance, dry asphalt pavement). The complete waveform clearly exhibits the zero-amplitude delay zone, primary echo wave packet, and successive attenuated reverberation peaks, which fully conform to the standard time-domain characteristics of air-coupled ultrasonic signals.

3. Figure S3

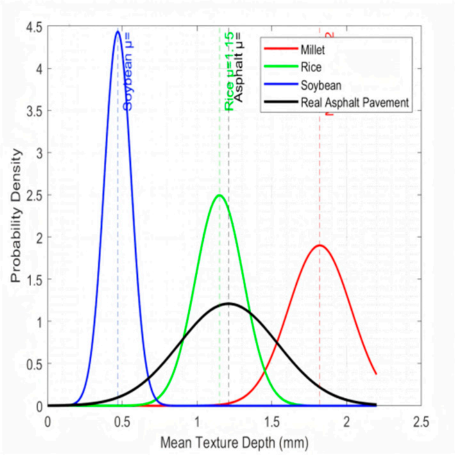

Figure S3. Fitted probability density distribution curves of mean texture depth (MTD) for millet, rice, soybean granular samples and three types of real asphalt pavements measured in Changchun. Vertical dashed lines mark the average MTD of each group, intuitively demonstrating that the roughness range of stacked granular media matches the micro-undulation features of actual road surfaces.
